# “EXHALE”: exercise as a strategy for rehabilitation in advanced stage lung cancer patients: a randomized clinical trial comparing the effects of 12 weeks supervised exercise intervention versus usual care for advanced stage lung cancer patients

**DOI:** 10.1186/1471-2407-13-477

**Published:** 2013-10-14

**Authors:** Morten Quist, Seppo W Langer, Mikael Rørth, Karl Bang Christensen, Lis Adamsen

**Affiliations:** 1 The University Hospitals Centre for Health Research (UCSF), Copenhagen, Denmark; 2 Centre for Integrated Rehabilitation of Cancer Patients (CIRE), Copenhagen, Denmark; 3 Department of Oncology, Copenhagen University Hospital Rigshospitalet, 5073 Copenhagen, Denmark; 4 Department of Biostatistics, University of Copenhagen, Copenhagen, Denmark; 5 Institute of Public Health, University of Copenhagen, Copenhagen, Denmark

**Keywords:** Maximal oxygen uptake (VO_2_peak), Functional capacity, Exercise, Quality of life, Anxiety, Depression, Advanced lung cancer, Chemotherapy

## Abstract

**Background:**

Lung cancer is the leading cause of cancer death in North America and Western Europe. Patients with lung cancer in general have reduced physical capacity, functional capacity, poor quality of life and increased levels of anxiety and depression. Intervention studies indicate that physical training can address these issues. However, there is a lack of decisive evidence regarding the effect of physical exercise in patients with advanced lung cancer. The aim of this study is to evaluate the effects of a twelve weeks, twice weekly program consisting of: supervised, structured training in a group of advanced lung cancer patients (cardiovascular and strength training, relaxation).

**Methods/Design:**

A randomized controlled trial will test the effects of the exercise intervention in 216 patients with advanced lung cancer (non-small cell lung cancer (NSCLC) stage IIIb - IV and small cell lung cancer (SCLC) extensive disease (ED)). Primary outcome is maximal oxygen uptake (VO_2_peak). Secondary outcomes are muscle strength (1RM), functional capacity (6MWD), lung capacity (Fev1) and patient reported outcome (including anxiety, depression (HADS) and quality of life (HRQOL)).

**Discussion:**

The present randomized controlled study will provide data on the effectiveness of a supervised exercise intervention in patients receiving systemic therapy for advanced lung cancer. It is hoped that the intervention can improve physical capacity and functional level, during rehabilitation of cancer patients with complex symptom burden and help them to maintain independent function for as long as possible.

**Trial registration:**

http://ClinicalTrials.gov, NCT01881906

## Background

Lung cancer is the leading cause of cancer death in North America and Western Europe [1]. In Denmark, the relative survival 1, 3 and 5 years after diagnosis of lung cancer is 32%, 13% and 10%, respectively. The best prognosis is achieved for patients who have lung cancer at an early stage (Non-small Cell Lung Cancer (NSCLC) stage I-IIIa and Small Cell Lung Cancer (SCLC) limited disease (LD)) and are receiving treatment with curative intent. For patients with advanced lung cancer (NSCLC IIIb-IV SCLC ED), the median survival after diagnosis is 10–13 months [2]. Despite improved treatment methods with surgery, chemotherapy, biological therapy and radiotherapy and more focus on supportive therapy, survival has not changed significantly over the last 10 years [3].

Patients with lung cancer receiving chemotherapy often experience a range of treatment- and disease-related symptoms such as dyspnea, cough, pain, decreased appetite, decreased functional capacity, and fatigue [4]. A comparison of quality of life (HRQOL) in treated lung cancer patients with other treated cancer patients indicates that patients with lung cancer in particular are suffering from several physical and psychosocial problems [5-7]. A Danish study highlighted physical, psychological and social problems among cancer patients with a broad spectrum of diagnoses. The results showed that lung cancer patients had more symptoms and side effects, increased anxiety and depression levels and impaired HRQOL compared to patients with 10 other cancer diagnoses [8]. Patients with metastatic, incurable tumors, such as lung and pancreatic cancer, reported the heaviest symptom and side effect burden compared with other cancer diagnoses [9].

It is well documented that physical activity can relieve symptoms and side effects in selected groups of cancer patients, including fatigue, cachexia and depression [10-15]. The benefits of physical activity for cancer patients have been described in several studies [16-21]. The majority of these studies include patients with breast cancer, prostate cancer and haematological malignancies. These studies have primarily measured the physical, functional capacity and HRQOL and predominantly included patient groups with relatively low disease burden.

In 2006 [22], our group demonstrated in a non-randomized study that 6 weeks of intervention with physical training (4× weekly) was beneficial to cancer patients with different diagnoses, both in early and in advanced stages. Patients increased their muscle strength (1RM) by 41% and V0_2_max by 14.5%. The effect of this intervention was confirmed in a randomized controlled study from 2009 [23], where in addition to an increase in muscle strength and V0_2_max, patients also reported a clinical significant reduction in fatigue. The study included 269 patients with different diagnoses, with few patients having lung cancer (n = 10). Analyses showed that lung cancer patients receiving chemotherapy indicated the same (percentage) physical progress as other cancer patients. However, lung cancer patients had significantly lower VO_2_max at baseline compared to other diagnostic groups (lung _VO2_max baseline = 1.15 L/min vs. other diagnoses baseline 2.27 L/min). Moreover, the lung cancer patients indicated that the exercise intensity (4 times per week) was too strenuous.

The existing knowledge about the effects of physical activity for lung cancer patients with advanced disease is sparse. The majority [24-28] of the studies that have tested aerobic training have been performed on lung cancer patients in an early stage of disease (NSCLC I-II LD SCLC) who did not receive chemotherapy and/or radiotherapy. The studies showed significant successes with regard to HRQOL and physical function (6 MWD) and physical capacity (VO_2_peak and 1RM) but have included relatively few patients (N = 10–23). Three studies have included patients with advanced inoperable lung cancer receiving chemotherapy [29-31]. Temel et al. [30] studied the effect of an eight-week (2 × weekly) training (fitness and strength) on 25 patients with lung cancer (NSCLC stage IIIb - IV), of which 18 patients received chemotherapy. The results showed no significant improvement in quality of life, anxiety or depression as measured by Functional Assessment of Cancer Therapy - Lung (FACT-L) and Hospital Anxiety and Depression scale (HAD). The physiological results showed a significant increase in strength of a single muscle group. There was no significant progress in the functional walk test (6MWD). Other studies have shown that physical exercise and relaxation techniques can relieve side effects and symptoms in cancer patients with advanced disease receiving chemotherapy [23,32,33].

In a pilot study from 2011 [29], our group showed that advanced (inoperable) lung cancer patients (NSCLC IIIb-IV SCLC ED) in chemotherapy could increase their exercise capacity (fitness, strength), functional capacity (6MWD) and emotional well-being (FACT-L) in a physical intervention (supervised and home exercises) two times weekly for 6 weeks. Patients reported no change in HRQOL. This study also showed that this intervention was feasible and safe for inoperable lung cancer patients receiving chemotherapy. Home training in addition to the supervised training was not feasible due to lack of compliance [34].

According to published data, patients with lung cancer in general have reduced physical capacity, functional capacity, poor quality of life and increased levels of anxiety and depression. Intervention studies indicate that physical training can address these issues in lung cancer patients with low stage (I-IIIa). We and others have shown that physical exercise for lung cancer patients with advanced stage (IIIb-IV) is safe and feasible (29;34). However, there is a lack of decisive evidence regarding the effects of physical exercise.

The aim of this study is to evaluate the effect of twelve weeks of a physical and psycho-social program consisting of: supervised, structured training in a group of advanced lung cancer patients (cardio and strength training, relaxation training) twice weekly. Primary outcome is (VO_2_peak). Our hypotheses are that patients who undergo this intervention will increase maximal oxygen uptake (VO_2_peak), strength (1RM), functional capacity (6MWD) and quality of life (HRQOL) and reduce the level of anxiety and depression.

## Methods/Design

This randomized controlled trial is being led by the University Hospitals Centre for Health Research at the University Hospital of Copenhagen, Denmark. The study is supported by grants from The Center for Integrated Rehabilitation of Cancer patients (CIRE), a center established and supported by The Danish Cancer Society and The Novo Nordisk Foundation. The study is prospectively registered with the Clinicaltrials.cov; registration number NCT01881906. Ethics approval has been obtained from the scientific Ethics Review Committee for the Capital Region of Denmark (J. HA-2008-06). The study is approved by the Danish Data protection agency Inspectorate (J. 2008-41-2279). The Intervention components will be tested on 216 patients with lung cancer (NSCLC stage IIIb - IV and SCLC ED) recruited from the lung cancer section at the dept. of oncology Copenhagen University Hospital, Rigshospitalet. All included patients will provide signed informed consent prior to initiation of any study procedures.

### Procedures

**Figure 1:**
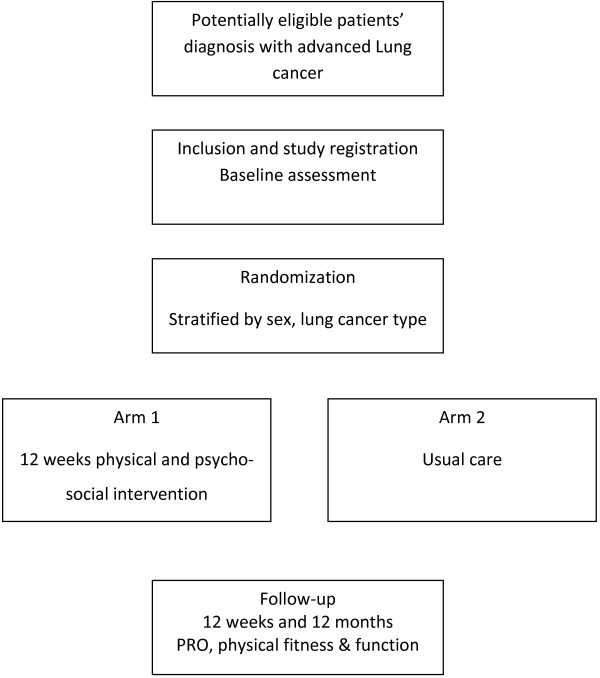
Study flowchart.

Eligible patients >18 years with a WHO performance status 0–2 with stage IIIb-IV NSCLC and SCLC-ED who are undergoing chemotherapy at the Department of Oncology are randomized to standard care or a 12 week physical and psycho-social intervention (Figure 1). Randomization is stratified for sex and lung cancer type (NSCLC or SCLC). Randomization is performed by the Copenhagen Trial Unit (CTU). Exclusion criteria are: brain or bone metastases; prolonged bone marrow suppression; anti-coagulant treatment; symptomatic heart disease, including congestive heart failure, arrhythmia or myocardial infarction diagnosed within the last three months; and inability to provide informed consent.

### Control – usual care

Patients who are randomized to the control group receive no training but are offered participation in the supervised training after completion of antineoplastic treatment, starting no earlier than twelve weeks after initiation of chemotherapy. Patients in early 2nd line treatment (“switch maintenance”) will be offered training after 12 weeks, although they have not necessarily completed chemotherapy.

### Intervention

The supervised training is carried out in groups of 12–16 patients and each session has a duration of 1.5 hours, is administered twice weekly and is supervised by a research physiotherapist. The training comprises warm up exercises, strength and fitness training as well as stretching. Warm up exercises consist of 10 minutes of light, stationery cycling, adjusted to 60-90% of the patient’s maximum HR. Strength training is carried out using 6 machines (Technogym: Leg press, chest press, lateral machine, leg extension, abdominal crunch, and lower back). The practical aim of strength training is to complete 3 series of 5–8 sets, with 70-90% of 1RM. The exercises are specifically selected to involve the largest possible number of muscle groups in the least number of exercises. To ensure progression in strength training, each patient is instructed in carrying out the 1RM test using each of the above-mentioned strength training machines once every second week, after which their program will be adjusted. Cardiovascular training is carried out as interval training on stationery bikes. Intensity is equivalent to 85-95% of each patient’s maximum HR and lasted approximately 10–15 minutes. After the training session, 5–10 minutes are dedicated to stretching the large muscle groups in order to increase agility. Following each training session, progressive relaxation of 15–20 minutes is performed.

### Pre training screening

Each patient is screened by a clinical nurse specialist prior to participating in each physical training session and before the physiological tests [35]. If one of the following criteria are met, the patient is prohibited from exercising/being tested on that day: diastolic blood pressure <45 or >95, heart rate (HR) at rest >115/min, temperature > 38°C, respiratory rate at rest >30/min, infection requiring treatment, fresh bleeding, total leucocyte count <1.0 10^9^/L or platelets <50 10^9^/L. Physical tests and HRQOL evaluation are performed at baseline and after six weeks of training.

### Study endpoints and assessments

Outcomes will be assessed at baseline (prior to randomization), 12 weeks and 12 months (Table 1). Baseline assessments involve a medical history; disease and treatment status is recorded from medical records and subjects will complete patient reported outcome (PRO) questionnaires, aerobic capacity (VO_2_peak (Cardio-Pulmonary Exercise Test (C-PET)), muscle strength (1RM), Functional capacity (6 Minute Walk Test (6MWT)).

**Table 1:** Assessments


*Physical capacity:*	Baseline
VO_2_*peak*	12 weeks, and 12 months from the start of the intervention
*Muscle strength:*	Baseline
1RM	12 weeks, and 12 months from the start of the intervention
*Body Composition:*	Baseline
weight / height, blood pressure, resting heart rate	12 weeks, and 12 months from the start of the intervention
*Lung function*	Baseline
Fev1	12 weeks, and 12 months from the start of the intervention
*Socio-demographic*	Baseline
self-developed	12 weeks, and 12 months from the start of the intervention
*Social support and network*	Baseline
MSPSS	12 weeks, and 12 months from the start of the intervention
*Health related quality of life*	Baseline
EORTC QLQ-C30-LC13, FACT-L HADS, SF36	12 weeks, and 12 months from the start of the intervention

### Primary and secondary endpoints

The primary endpoint will be VO_2_ peak, as assessed with an aerobic capacity incremental C-PET on a cycle ergometer (Monark, ergomedic 839E). The C-PET is carried out by a physiotherapist who is blinded to the patient’s study group allocation. The test consists of a warm-up phase 2–4 minutes of cycling at a sub maximal load (10–50 watts). After the warm-up period the load increases after a short break (<2 minutes) by 5–10 watts every minute, until exhaustion or a possible symptom limitation (e.g. dizziness, sudden pain, vomiting sensation). Expired gases are analyzed continuously by a metabolic breath-by-breath analysis and calculated as an average over 15 seconds using the Oxycon Pro, Jaeger measurement system. During the C-PET, oxyhemoglobin saturation and heart rate is continuously monitored. After each test, maximum ventilation, respiratory exchange ratio (RER), possibly plateau in the increase in VO2, self-perceived exertion perception in the final seconds of the C-PET and maximal heart rate (Polar Team System 2, Polar, Finland) are recorded. Rating of perceived exertion is evaluated at the end of each workload using the modified Borg Scale. The primary outcome will be a comparison of VO_2_ peak in the intervention and control arms at the conclusion of the intervention (i.e. at the 12-week assessment).

Muscle strength is measured by the one repetition maximum (1RM) [36] test using a Technogym™ that includes a leg press (lower extremity), chest press (pectoral muscles), lateral machine (latissimus dorsi), leg extension (quadriceps femoris), abdominal crunch (rectus abdominis) and lower back press (erector spinae). The 1RM test is the golden standard and has been found to be a reliable assessment to measure upper and lower extremity strength [36].

Functional capacity is measured by a 6 MWD test. The test is carried out over a pre-measured distance of 28 meters, in compliance with the American Thoracic Society (ATS) statement [37]. The 6MWD test has demonstrated good reliability and validity in COPD patients who are similar to patients with lung cancer with regard to disease patophysiology and symptomatology [38].

Lung capacity Forced Expiratory Volume in 1 second (FEV1) is measured using a standard spirometry in a standing position with the use of the Oxycon Pro, Jaeger measurement system.

Patient reported Outcomes (PRO’s): PRO’s include standard validated questionnaires assessing general wellbeing using the 36-Item Short Form Health Survey (MOS SF-36) [39], quality of life using the European Organization for Research and Treatment of Cancer Quality of Life Questionnaire (EORTC QLG C-30 + LC13) [40] and the FACT instrument, comprising two parts, i.e. the general part (FACT-G) and the lung specific part (FACT-L) [41], social support and network using the Multidimensional Scale of Perceived Social Support (MSPSS) [42]. Furthermore sleep quality will be assessed using the Pittsburgh Sleep Quality Index [43], and levels of anxiety and depression will be assessed using the Hospital Anxiety and Depression Scale (HADS) [44]. Additional questionnaires will assess self-reported physical activity, labour market attachment, work ability and social reintegration. Demographic data is collected from self-developed questionnaires and training diaries.

### Statistical analysis

#### Sample size

The sample size calculation for the primary outcome VO2peak is based on earlier data [29] in which 55 patients through six weeks of training achieved an increase of 0.85 ml/kg/min (SD = 2,48 ). It is assumed that patients in the control group of the current study will have a reduction of 0.5 ml/kg/min for VO2peak and thus a total of 108 patients (54 in each arm) will be sufficient to achieve a power of 80% (risk of type 2 error set at 0.20) using a significance level of 0.05 (risk of type 1 error set at 0.05). We expect a drop-out rate of 50% and therefore another 108 patients must be included yielding a sample size of 216 patients.

Data entry is done using OpenClinica and statistical analysis will be performed using Statistical Analysis Systems (SAS) version 9.2. The statistician will prepare results without knowledge of the randomization coding. The primary endpoint will be reported as a two-sample t-test comparing change scores in the two randomization groups. The patient reported outcomes will be reported as either means with corresponding 95% confidence limits or as medians interquartile range (IQR) for continuous data. Categorical data will be reported as proportions and compared across randomization groups using chi-squared tests. Significance level is set at 0.05.

## Discussion

Potential beneficial effects of physical exercise for cancer patients remains to be demonstrated for lung cancer patients with advanced disease. Advanced lung cancer is incurable, and is the leading cause of cancer deaths world-wide. The aim of the treatment is to improve QoL and prolong life for these patients, therefore interventions with a focus on VO_2_peak and functional capacity are much needed as physical exercise has shown to have a positive impact on QoL It has been shown [45] that patients with advanced stage lung cancer significantly lowered their functional capacity (6 MWD) after two series of chemotherapy. Moreover, patients with low functional capacity before starting chemotherapy had significantly more disease progression and significantly shorter lifespan, compared to those with a higher functional capacity. Another study [46] supports this result and found that functional capacity is a strong independent predictor of survival in advanced NSCLC that adds to the prediction of survival beyond traditional risk factors, which may improve risk stratification and prognostication in NSCLC.

Physical exercise for advanced stage lung cancer patients is a new and unexplored area. The present intervention has the potential to change standard care with a simple, safe, relatively inexpensive physical exercise intervention that could improve the QoL and reduce symptoms and side effects for patients with advanced stage lung cancer. This group of patients does not receive curative treatment and is often not offered meaningful rehabilitation. This means that, for several months, the patients are not offered regular exercise despite reduced functional capacity, impaired QOL and burdensome side effects. The results from this study may help to elucidate any positive effects of physical exercise during chemotherapy for lung cancer patients with advanced disease, and whether it is possible to improve physical capacity and functional level, in the rehabilitation of cancer patients with complex symptom burden not receiving treatment with curative sight, and help them to maintain independent function for as long as possible.

## Conclusions

The current exercise intervention has already proven to be safe and feasible for advanced stage lung cancer patients, with a high completion and adherence rate (<70%) and of without adverse reactions.

The present randomized controlled study will provide additional data on the effectiveness of a supervised exercise intervention in patients receiving systemic therapy for advanced lung cancer, hopefully contributing to an improvement of the overall treatment results.

## Competing interests

The authors declare that they have no competing interests.

## Authors’ contributions

MQ, SL, MR and LA devised the study concept and design. All authors contributed to the study protocol. KBC was responsible for overseeing the statistical section. MQ, SL, MR, KBC and LA wrote the manuscript. All authors read and approved the final manuscript.

## Pre-publication history

The pre-publication history for this paper can be accessed here:

http://www.biomedcentral.com/1471-2407/13/477/prepub
